# Lifestyle interventions to prevent gestational and type 2 diabetes among migrant women from low- and middle-income countries: a systematic review

**DOI:** 10.1080/16549716.2026.2658925

**Published:** 2026-04-17

**Authors:** Blessing Jaka Akombi-Inyang, Shirin Mumu, Azeb Gebresilassie Tesema, Dafna Merom

**Affiliations:** a School of Population Health, University of New South Wales, Sydney, Australia; b School of Medicine, Western Sydney University, Penrith, Australia; c School of Health Sciences, Western Sydney University, Penrith, Australia

**Keywords:** Physical activity, dietary habits, maternal health, health disparities, cultural adaptation

## Abstract

Migrant women from low- and middle-income countries (LMICs) living in high-income settings experience disproportionately high risk of gestational diabetes mellitus (GDM) and type 2 diabetes mellitus (T2DM). This review aimed to identify and synthesise culturally adapted lifestyle interventions for preventing or managing GDM and T2DM among migrant women from LMICs, focusing on intervention components, cultural adaptation strategies, and behavioural and metabolic outcomes. Five databases (PubMed, Embase, Scopus, CINAHL, Cochrane Central) were searched using Preferred Reporting Items for Systematic reviews and Meta-Analysis 2020 guidelines. Eligible studies included experimental designs involving lifestyle interventions delivered to migrant women from LMICs in high-income countries, reporting outcomes related to GDM or T2DM targeting behaviour change. Data were synthesised narratively; study quality was appraised using RoB2 for RCTs and a structured narrative approach for non-randomised designs. Certainty of evidence was evaluated using GRADE. Eight studies met the inclusion criteria. Sample sizes ranged from 28 to 641 participants. Intervention duration varied from 6 weeks to 12 months. Most interventions incorporated atleast one culturally tailored component, such as bilingual delivery, culturally adapted dietary education, or community-based engagement. Improvements were reported across dietary behaviours, physical activity, glycaemic measures, or diabetes-related knowledge; however, effect sizes were modest and inconsistent. Interventions combining dietary modification, physical activity, and culturally adapted delivery demonstrated greater improvements than exercise-only or digital-only programmes. Overall certainty of evidence ranged from low to moderate. Culturally adapted, multi-component lifestyle interventions show promise for improving behavioural and metabolic outcomes among migrant women from LMICs; however, the evidence base remains limited.

## Paper Context

**Main findings:** Culturally adapted, multi-component lifestyle interventions show modest but inconsistent improvements in behavioural and metabolic outcomes among migrant women from low- and middle-income countries with overall low-to-moderate certainty of evidence.

**Added knowledge:** This review highlights cultural adaptation as a key feature of intervention effectiveness and identifies important evidence gaps, including limited high-quality studies and challenges in comparing heterogeneous interventions.

**Global health impact for policy and action:** Policies should prioritise scalable, culturally responsive interventions – such as bilingual and community-based approaches – to reduce diabetes disparities and improve maternal health outcomes in migrant populations.

## Background

Type 2 diabetes mellitus (T2DM) and gestational diabetes mellitus (GDM) are significant public health concerns, with their prevalence increasing globally [1–3]. The 10th edition of the International Diabetes Federation (IDF) reported that diabetes, where T2DM accounts over 90% of it – is one of the emerging global emergencies, with more than half a billion people living with the condition worldwide in 2021 [3]. GDM, characterised by elevated blood glucose levels that develop or are first identified during pregnancy, may occur at any point in the antenatal period [3]. It is generally expected to resolve after childbirth but increases the risk of future T2DM in both the mother [4] and the child [5]. Globally, the prevalence of GDM ranges from 5% to 16%, varying based on the population and the screening and diagnostic criteria applied [3,6].

While both conditions are rising globally, the burden remains disproportionately higher among people living in low- and middle-income countries (LMICs) [1–3]. This variation is confounded by significant demographic, socioeconomic, and geographic disparities, as well as lack of access to care and follow-up services. Additionally, ethnicity or country of origin has been identified as one of the risks for GDM and T2DM [6–8].

Migrant women from LMICs in developed countries are a particularly vulnerable group, not only due to genetic susceptibility but also due to lifestyle and cultural changes resulting from migration, including acculturation stress, dietary transitions towards highly processed foods, reduced opportunities for physical activity, and socioeconomic and healthcare barriers, such as financial instability, language barriers, and poor health literacy [8]. These factors collectively hinder timely diagnosis, engagement in lifestyle modification, and access to appropriate care [8]. Pregnancy represents a critical and time-sensitive window in which behavioural interventions may reduce the immediate risk of GDM and influence longer-term metabolic trajectories that prevent progression to T2DM [9].

GDM and T2DM are distinct conditions with different diagnostic pathways and clinical implications, yet they share common modifiable behavioural and metabolic risk factors, such as diet quality, physical inactivity, overweight/obesity, and insulin resistance [9]. While acknowledging these differences; however, this review focuses on prevention rather than treatment, emphasising lifestyle interventions that target shared determinants of dysglycaemia among migrant women from LMICs living in high-income countries who are at risk of developing either condition. A previous systematic review by Sagastume et al. [10] have acknowledged these commonalities and explored the effects of 48 lifestyle interventions delivered in LMICs on the incidence of T2DM (*n* = 45 RCTs) and GDM (*n* = 3 RCTs) and cardiometabolic indicators. The review confirmed that lifestyle interventions had significant effect in reducing the incidence of both T2DM and GDM. In addition, significant effects were shown on glycated haemoglobin (HbA1c), fasting glucose, and anthropometric indicators for both T2DM and GDM [10]. Despite these positive reports in the general population, existing strategies often fail to consider the unique cultural, social, and economic contexts of migrant women from LMICs, limiting our knowledge about the effectiveness and scalability of these interventions in this population [8]. Addressing these gaps requires a deeper understanding of the underlying factors and the design of tailored approaches to improve health outcomes for migrant mothers and their offspring [8,11].

Current evidence is limited in its ability to inform culturally responsive and context-specific strategies tailored to this high-risk group [12,13]. In this review, culturally responsive interventions are conceptualised as approaches that actively incorporate participants’ cultural beliefs, values, preferences, communication styles, health practices, and community structures into the design and delivery of lifestyle programmes [14–16]. Such interventions may include the use of bilingual health workers, culturally tailored educational materials, community-based delivery models, or adaptation of dietary and physical activity components to align with cultural norms. Such alignment enhances acceptability, trust, and sustainability, thereby improving engagement, adherence, and ultimately health outcomes for migrants in general, but particularly for women who experience multiple layers of marginalisation [14–16]. A systematic review by Rawal and colleagues [17] examined the effectiveness of lifestyle interventions to manage T2DM among migrants and ethnic minorities of both genders living in industrialised countries; the importance of developing culturally appropriate approaches have been highlighted in their conclusion [17] but the specific cultural aspects of the successful interventions were not systematically identified and discussed. Till date, no prior review has specifically synthesised lifestyle interventions targeting both GDM and T2DM among migrant women from LMICs living in high-income countries, a population whose risk pathways, sociocultural contexts, and barriers to care differ substantially from general migrant or indigenous populations.

This systematic review therefore aims to identify and synthesise culturally adapted lifestyle interventions for the prevention or management of GDM and T2DM among migrant women from LMICs living in high-income countries, with a particular focus on their key components, cultural adaptation strategies, and associated behavioural and metabolic outcomes. By characterising key intervention components, cultural adaptation strategies, and metabolic and behavioural outcomes, this review provides a clearer evidence base to support future practice and policy, ultimately contributing to more equitable and culturally sensitive maternal health strategies.

## Method

Because the available intervention studies varied substantially in populations (pregnant versus postpartum women), outcomes (GDM incidence, glycaemic markers, and behaviour change), and intervention timing, we synthesised the findings narratively. Given this heterogeneity in study design, populations, timing, and outcomes, direct comparisons across studies are not appropriate and findings should be interpreted within the context of each individual study.

Outcomes were coded according to whether studies reported GDM-specific or T2DM-related endpoints. This approach allowed us to examine GDM and T2DM prevention as related – but non-interchangeable – constructs connected through shared lifestyle-related risk factors. During synthesis, findings were interpreted within the appropriate clinical context for each condition, and where studies addressed only one clinical endpoint, results were considered within that specific context without extrapolating across conditions.

The reporting of the systematic review adhered to the Preferred Reporting Items Systematic Reviews and Meta-Analysis (PRISMA) guidelines [18]. The protocol is available at PROSPERO [CRD42024589416].

### Search strategy

A comprehensive search was conducted across five electronic databases – PubMed, Embase, Scopus, CINAHL, and the Cochrane Central Register of Controlled Trials – to identify relevant studies examining lifestyle interventions aimed at preventing or managing GDM and T2DM among migrant women from LMICs living in high-income countries. The search strategy combined Medical Subject Headings (MeSH), Emtree terms, and free-text keywords related to diabetes, lifestyle interventions, pregnancy, migration, and LMIC populations. Terms were linked using Boolean operators (‘AND’ and ‘OR’) and adapted for each database to ensure maximal sensitivity.

The search was guided by the PICO framework and adhered to its registered protocol. In addition to database searches, we manually screened the reference lists of all included studies and conducted forward citation tracking using Google Scholar to identify any additional eligible publications.

Consistent with the registered protocol, the search was restricted to peer-reviewed published articles; grey literature sources and clinical trial registries were not included. Given the focus of this review on lifestyle intervention studies with clearly defined methodologies and outcome measures, restricting the search to published academic literature ensured methodological consistency and alignment with comparable systematic reviews in this field. No date limits were applied, and only studies published in English were eligible. The full search strings used for each database, along with the date of the final search, are provided in Supplementary Table S1.

### Inclusion and exclusion criteria

The eligibility criteria were defined using the PICO framework. The population comprised migrant women born in LMICs who are currently residing in high-income countries. This included pregnant women at risk of, or living with GDM, as well as postpartum or adult women at risk of, or living with, T2DM. The intervention comprised lifestyle modifications, including nutritional strategies such as nutritional education, dietary consultation, improving physical activity levels, reducing sedentary behaviour, or a combination of these approaches. The comparison was standard or usual care or placebo for at-risk populations or nothing at all for the general population. The main outcome was GDM and/or T2DM as measured by the study and/or behavioural outcomes, such as a change in diet, physical activity behaviour and other antecedent to behaviour change including motivation, knowledge, and self-efficacy amongst others. Study types included randomised controlled trials (RCTs), quasi-experimental designs/non-RCTs, and pre-post evaluations without a comparison group/pre- and post-studies. Observational studies were excluded. Only articles published in English were eligible, with no limitations on the publication date.

### Study selection

All retrieved studies were imported into Covidence, where duplicate records were removed. An initial screening was then conducted based on article titles and abstracts. In the final screening phase, the full texts of retrieved articles were reviewed for relevance, and only those meeting the inclusion criteria were retained. During screening, studies were included only if the sample comprised women born in LMICs residing in high-income countries, in alignment with the clarified population definition. The screening process was conducted independently by two reviewers (SD, BAI), with any disagreements resolved through discussion and consensus with other co-authors (DM, SJM).

### Data extraction

Data was extracted using a customised template that captured key study characteristics, including author, year, country, study design, sample size, participant characteristics, intervention components, comparator (where applicable), diabetes-related outcome, and behavioural outcome as shown in Table 1. In addition, we extracted information on cultural adaptation strategies based on the five categories described by Henderson and See [16], as well as health-promotion principles relating to co-design, capacity building and empowerment as shown in Table 2.

**Table 1.** Characteristics of included studies.

Source & Study design	Population	Intervention	Comparator	Outcomes	Results
Wieland [2024] RCT [19]	Country: USA Migrants: Hispanic or Latino Sample: 370 women diagnosed with T2DM Mean age:53	The same usual care as the comparator group PLUS the digital storytelling intervention. Participants watched a 12-minute culturally sensitive video containing four stories, and a closing educational message reinforcing the four diabetes self-management behavioral goals (healthful diet, physical activity, medication adherence, glucose self-monitoring). The video was watched in a private room with study staff present. Five monthly automated text messages were sent to participants asking to rate their self-efficacy and motivation to control their diabetes. Those scored < 7 were encouraged to view the video again.	Usual clinical care for diabetes and two cards describing how to engage healthcare teams and access diabetes-related resource	Primary outcome: HbA1c level Secondary outcomes Biometry: BMI blood lipids and blood pressure Diabetes self-management survey (SDSCA) Diabetes self-efficacy scale	Haemoglobin A1c level mean (SD), % Intervention: 9.1 (1.7) reduced to 8.4% (1.6) Control: 9.4 (1.8) reduced to 8.8% (2.0) *p* = 0.04 Intervention group were 50% more likely to have reduced Haemoglobin A1c (*p* = 0.06)
Wikström [2021] Pre-post (no comparator) [20]	Country: Finland Migrants: Somali Sample: 18 women at risk of T2DM Mean age: 47 ± 10	***The StopDia lifestyle counselling*** Six group meetings of 1.5 hour and the BitHabit healthy lifestyle support mobile application. Homework and exercises using the participant’s workbook, such as keeping a diary of their physical activities or fruit and vegetable consumption.	Not applicable	Primary outcome: Anthropometry & blood pressure Secondary outcomes: Dietary & Physical activity changes Capable to make changes in diet; Vegetable consumption; Fruit and berries consumption; Eating breakfast, lunch or dinner. Physical activity: Capable to increase physical activity; Incidental exercise three or more times per week; Planned exercise three or more times per week; Steps per day.	Pre-post changes: Weight, (kg): 91.1 ± 12.8; 91.2 ± 13.3 (Not significant) Waist Circumference, (cm) 106.2 ± 94; 105.5 ± 11.3 (Not significant) Pre % post % Capable to make changes in diet: 76%; 57% Vegetables daily: 50%; 80%, *p* = 0.016 Fruit % daily: 52%; 52% Breakfast three times or more per week: 91%; 9%5 Capable to increase physical activity: 77%; 91% Incidental exercise ≥ 3 times/week: 48%; 86% Planned exercise ≥ 3 times/week: 23%; 48% Steps/day 3771 ± 2866; 4568 ± 2080
Melero [2020] RCT (two comparison groups) [24]	Country: Spain Migrants: Hispanic Sample: Women at their 8–12 weeks of gestation and high risk of GD Age Intervention: 31.7 ± 5.4 Comparator: 31.2 ± 5.6	***Nutritional therapy*** Nutritional therapy based on MedDiet principles, with EVOO and pistachio supplements. At visit 1 (12–14 GW): Pregnant women were advised to increase their consumption of extra virgin olive oil (EVOO) (≥40 mL/day) and have a handful of pistachios (25–30 g) at least 3 days a week. At visit 2 (24–28 GW): meeting of approximately 2 h with a dietician. MedDiet nutritional advice was reinforced, and participants were told to increase their consumption of EVOO and nuts. 10 L of EVOO (about 1 L each 10 days) and two g of roasted pistachios (about 160 g a week) were provided at no cost at visits 1 and 2 to ensure the consumption.	*Comparator 1*: Visit 1: advised to restrict fat intake, with the consumption of EVOO limited to a maximum of 40 mL/day, and nuts < 3 days per week as usually recommended. Visit 2: were asked to limit fat intake. *Comparator 2*: An extension to the RCT included a Real-World group comprising a cohort of Hispanic women assessed at 8–12 weeks’ gestation, who were given similar advice as Comparator 1.	Primary outcomes: i) Incidence of GDM ii) fasting serum glucose (glucose oxidase). iii) HbA1c (%) level. Secondary outcomes Changes in diet and physical activity scores throughout pregnancy	Intervention: 19 (14.8%); Control 1: 34 (25.8%). p = 0.021, HbA1c (%) at 24–28 week: Intervention: 5.0 ± 0.3; Control 1: 5.1 ± 0.3, p = 0.021 HbA1c 36–38 weeks: Intervention: 5.3 ± 0.2; Control 1: 5.5 ± 0.3, p = 0.001 Fasting serum Insulin: No difference, *p* = 0.87 MedDiet Score: Intervention: 7.5 ± 1.6 Control1: 5.0 ± 2.0 *p* = 0.034 Physical activity score: No difference, *p* = 0.299
Kandula [2016] Pre-post (no comparator) [25]	Country: USA Migrants: South Asians Sample: i) Women with children between the ages of 6–14 years; ii) Had one or more risk factors for developing DM iii) A personal history of GDM. Mean age: 40 ± 5	***Exercise intervention*** Every week, participants were required to attend a minimum of two out of three exercise classes. Certified exercise instructors conducted classes at Metropolitan Asian Family Services (MAFS) and Ultimate Martial Arts (UMA). Instructors led participants in 45 min of moderate-intensity exercise drawing on Zumba and aerobics.	Not applicable	Secondary outcomes: − HbA1c levels.	There was a small statistically significant change in HbA1c: Pre and post intervention mean change: 0.1 [95%Cl: 0.00, 0.12]
Telle-Hjellset [2013] (Main trial) RCT [26] *Helland-Kigen [2014] [24] *Råberg Kjøllesdal [2011] [25] *Johansen [2010] [26]	Country: Norway Migrants: Pakistani living in Oslo Sample: 198 women at no risk of diabetes Age: 25–62 years Mean age: 42.7	***InnvaDiab-DE-PLAN*** Combination of group sessions, individual counselling & organised exercise groups: Six group sessions 2-hour each, focused on the importance of diet and physical activity for blood glucose regulation with culturally adapted audiovisual materials as aid. Dietary advice were given individually, based on their blood tests. Encouraged to walk for one hour twice a week throughout the trial period.	Feedback provided on blood sugar levels and received lifestyle advice in one single (short version) group session after the follow-up tests.	Main outcomes: Telle-Hjellset paper: Fasting and 2-hour blood glucose −Fasting levels of insulin Helland-Kigen paper: Dietary Behaviour change Råberg Kjøllesdal paper: Perception of risk Johansen paper: Intake of specific drink and food	Fasting blood glucose and fasting insulin (between-group differences −0·16 mmol/l, *p* = 0·003 and −5·8 pmol/l, *p* = 0·036). Between group dif. Change type of fat (*p* = 0.001), increase vegetable intake (*p* = 0.001); reduce sugar (*p* = 0.003) Reduced soft drinks with sugar (*p* < 0.001); and deep fries (*p* = 0.001); increase use of Rapeseed oil *p* = 0.001

*Telle-Hjellset (2013) was the primary study from which Johansen (2010), Råberg Kjøllesdal (2011), and Helland-Kigen (2014) were derived.

**Table 2.** Cultural, health promotion principles and evaluation aspects of intervention delivered to migrant women to manage or prevent GDM and T2DM.

	Telle-Hjellset (2013)	Kandula (2016)	Melero (2020)	Wikström (2021)	Wieland (2024)
Participants and Location	Pakistani migrants in Norway (Oslo)	South-Asian (India & Pakistan) migrants in USA (Chicago)	Latin American migrants in Spain (Madrid)	Somali in Finland capital region	Hispanic migrants in USA (Minneapolis & Phoenix)
Intervention focus	Lifestyle education	Exercise & two sessions on diet (optional)	Mediterranean diet	Stop Diabetes Model (Lifestyle)	Diabetes-management & lifestyle
Format of delivery	9 Groups (10–12 women each)	Group exercise	Individual- during pregnancy follow-up visit	Group counselling	Individual digital delivery
Cultural components	Bilingual deliverer; Use of interpreter service; Use of culturally sensitive multimedia (e.g. video, figures, card)	Bilingual deliverer; Cultural competency training; Use of interpreter service; Establishing community point-of-care service	Establishing community point-of-care service	Bilingual deliverer; Cultural competency training; Use of culturally sensitive multimedia (video, figures, card); Establishing community point-of-care service	Bilingual deliverer; Cultural competency training; Use of culturally sensitive multimedia (video, figures, card); Establishing community point-of-care service
Co-design & capacity building/Empowerment	Yes	Yes	No	Yes	No
Intervention dose	Six sessions 2-hour each	3 weekly classes 1-hour each	4 visits at 8, 12, 24 & 36 gestational week; 2-hour	Six sessions 1.5-hour each	Storytelling 12 mins video & five monthly text prompts
Duration of intervention	7 months	4 months	9 months	3 months	6 months
Program deliverer professions	Female-only researchers who speak Norwegian and Urdu	Certified exercise instructors	Obstetricians, dieticians, diabetes nurse educators, and endocrinologists	Researchers with Somali background & Somali volunteers health care students	Language- and culture-congruent study staff working at the clinics
Attendance	60% attended 4 or more sessions	57% attended at least 80% of classes	89% of attended all visits	Mean participation was 50% 70% used mobile application	Not reported
Formative or process evaluation	Change in antecedent to behaviour change	Perceptions about the program	No evaluation	Satisfaction from the intervention	Satisfaction with intervention and perception of wellbeing

### Quality appraisal

The risk of bias in the included RCTs was assessed using the Cochrane Risk of Bias 2 (RoB2) tool [19], which evaluates potential bias across five key domains including randomisation process and allocation concealment, deviations from intended interventions, missing outcome data, measurement outcome and selection of reported results.

Two independent reviewers (SJM and DM) conducted the assessments, and any discrepancies were resolved through discussion until consensus was reached. Each study was categorised as having either low risk of bias, some concerns or high risk of bias.

For non-randomised and pre – post studies, the ROBINS-I tool [20] was not applied because several of the included studies lacked a comparison group, making ROBINS-I unsuitable for consistent appraisal across the different study designs. Instead, these studies were evaluated using a structured narrative appraisal focusing on methodological clarity, baseline characteristics (where reported), fidelity of intervention delivery, completeness of outcome data, and potential sources of bias, such as selection, performance, and detection bias. This approach aligns with recommended methods for appraising heterogeneous study designs in systematic reviews where validated tools cannot be uniformly applied. The quality assessment of these pre–post interventions is summarised narratively in the text.

In addition to the risk-of-bias assessment, we evaluated the certainty of evidence for the primary outcomes using the GRADE approach [21]. Certainty ratings were assigned at the outcome level and considered domains such as risk of bias, inconsistency, indirectness, imprecision, and publication bias. RCTs were initially rated as high-certainty evidence and downgraded where applicable, whereas non-randomised and pre–post studies were initially rated as low-certainty evidence. A summary of GRADE ratings for all relevant outcomes is presented in Supplementary Table S2.

## Results

**Figure 1.**
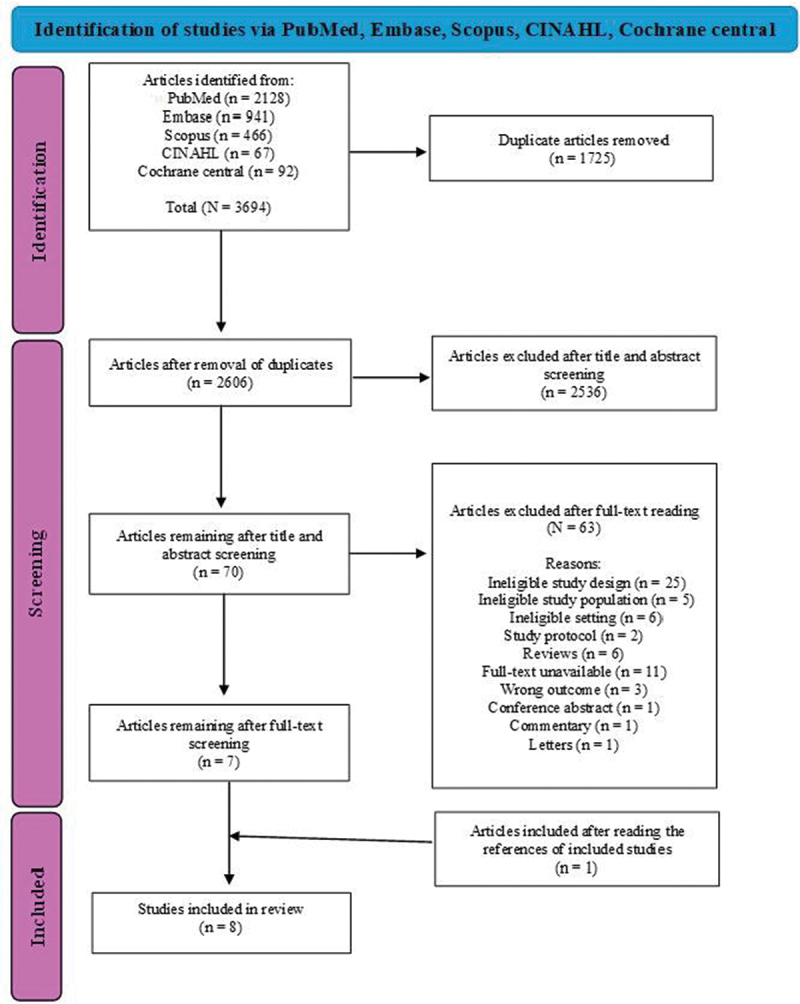
PRISMA 2020 flow diagram for study selection.

A total of 3,694 articles were retrieved from five databases. After removing duplicates, 2,606 articles remained. Screening of titles and abstracts excluded 2,536 articles. The full texts of the remaining 70 articles were assessed for eligibility, of which 63 were excluded for not meeting the criteria. Seven articles were included in the final review. A manual search of the bibliographic references of the retained articles identified one additional study, as shown in Figure 1.

## Characteristics of included studies

Eight studies were included in this review, comprising culturally adapted lifestyle interventions delivered to migrant women from LMICs residing in high-income countries, including Europe, the United States, and Australia. As shown in Table 1, the studies varied substantially in design (four RCTs, one non-randomised controlled study, and three pre – post studies), intervention intensity, cultural tailoring, and outcome measures. Sample sizes ranged from 20 to 641 participants, limiting comparability across studies. The interventions also differed in population diversity, scope, and design, as well as in the specific outcomes assessed, including dietary behaviours, physical activity, psychosocial measures, fasting plasma glucose, and other glycaemic indicators. To maintain conceptual clarity, the findings were presented separately for GDM-specific outcomes and T2DM-related metabolic outcomes.

### Population diversity and setting

Studies involved distinct demographic groups. In Norway, Telle-Hjellset (2013) [26] examined 198 Pakistani women (25–62 years) residing in Oslo. Kandula (2016) [25] recruited 30 South Asian women in the U.S., all with children aged 6–14 years and at least one diabetes risk factor (e.g. BMI ≥ 25 kg/m^2^, GDM history, or familial diabetes). Melero (2020) [24] in Spain included 600 pregnant women with normal fasting glucose at 8–12 gestational weeks to evaluate GDM prevention via diet. Wieland (2024) [22] focused on 451 U.S.-based Hispanic/Latino individuals (70% women) with poorly controlled T2DM (HbA1c ≥ 8%).

### Intervention scope and design

The interventions varied in intensity, cultural tailoring, delivery format, and behavioural focus. Strategies ranged from structured lifestyle education and culturally adapted nutrition counselling [26], group-based exercise programmes [25], Mediterranean diet interventions [24], and lifestyle counselling with mobile-app support [23], to culturally grounded digital storytelling modules [18]. This heterogeneity limits comparability across studies, and the included interventions should not be interpreted as directly comparable. Instead, the findings provide context-specific insights into culturally responsive strategies suited to migrant populations.

### Dietary behaviour outcomes

Seven studies [22–24,26–29] assessed dietary behaviour using validated tools or self-report, and across these interventions, dietary outcomes were generally more consistent and larger in magnitude than metabolic changes. Common improvements included increased fruit and vegetable intake (0.5–1.2 servings/day) and reduced refined carbohydrate consumption. Melero et al. (2020) [24] demonstrated improved diet quality (effect size d ≈ 0.32) through a culturally adapted Mediterranean diet programme. Interventions incorporating bilingual educators, culturally relevant materials, and community engagement typically yielded stronger dietary improvements [22–26]. Earlier studies such as Johansen (2010) [29] and Helland-Kigen (2014) [27] documented significant increases in intentions to reduce fat (*p* = 0.004; *p* = 0.001) and sugar intake (*p* = 0.001), alongside higher vegetable consumption (*p* = 0.003; *p* = 0.001). Participants also reduced sugary drink intake (*p* < 0.001) and adopted healthier cooking oils (*p* = 0.001), although behaviours, such as meat/fish consumption remained unchanged. However, while these culturally grounded educational programmes enhanced motivation and intention, sustained behaviour change was not always observed, highlighting important gaps between awareness and long-term dietary adherence.

### Physical activity outcomes

Across the included studies, physical activity (PA) outcomes showed mixed but generally positive patterns. Three interventions specifically targeted PA, with two reporting increases of 40–75 minutes per week in activity levels. Wikström (2021) [23], which combined lifestyle counselling with a mobile app, demonstrated substantial increases in both planned exercise (from 23% to 48%) and incidental activity (from 48% to 86%), alongside improvements in vegetable intake. However, despite these behavioural changes, weight and BMI remained stable (91.1 kg ±12.8 vs. 91.2 kg ±13.3; BMI 32.8 ± 4.3 vs. 32.9 ± 4.4), highlighting the difficulty of translating increased activity into measurable metabolic benefits over short periods.

Not all PA-focused interventions produced favourable metabolic effects. Kandula et al. (2016) [25]—a physical activity – only intervention – reported a slight increase in HbA1c (0.1%, 95% CI: 0.00–0.12), reinforcing evidence that PA alone may be insufficient without concurrent dietary modification. Pre – post studies also showed that increases in walking time tended to occur primarily among participants with higher baseline motivation, suggesting that motivational readiness plays a key role. Collectively, the findings indicate that while culturally sensitive PA interventions can improve engagement and activity levels, multicomponent models integrating both diet and PA are more likely to yield metabolic benefits.

### Psychosocial outcomes

Two studies assessed psychosocial outcomes such as diabetes knowledge, motivation, risk perception, and self-efficacy. Both reported meaningful improvements (effect sizes d = 0.20–0.40), with the strongest effects observed in interventions delivered by trained bilingual health workers – highlighting the importance of culturally congruent delivery.

Råberg Kjøllesdal (2011) [28] found increased recognition of physical inactivity (*p* = 0.020) and family history (*p* = 0.026) as diabetes risk factors following the intervention. However, persistent misconceptions regarding overweight, diet, and stress underscored the need for more targeted and culturally tailored education. Behavioural shifts facilitated through technology-assisted models, such as the mobile-app intervention in Wikström (2021) [23], also contributed to improved psychosocial readiness for change, even when metabolic outcomes remained modest. Overall, psychosocial outcomes were more consistently positive than metabolic indicators, suggesting that culturally responsive interventions may strengthen knowledge and motivation even when physiological outcomes require longer-term or more intensive intervention strategies.

### GDM-specific outcomes

One intervention focused explicitly on GDM prevention among pregnant women at high risk. Melero (2022) [24] evaluated a culturally adapted antenatal lifestyle programme incorporating nutrition counselling, physical activity promotion, and behavioural support. Women in the intervention group demonstrated a lower incidence of GDM compared with those receiving standard care (14.8% vs 25.8%). Improvements were also observed in gestational glucose regulation and HbA1c levels at both 24–28 weeks and 36–38 weeks of pregnancy. These findings suggest that culturally adapted antenatal interventions may be effective in reducing GDM risk during pregnancy.

### T2DM-related metabolic outcomes

Four studies targeted women with established T2DM or those at increased risk of developing T2DM outside pregnancy. Telle-Hjellset (2013) [26] reported significant improvements in fasting glucose and insulin resistance following a culturally tailored lifestyle programme promoting healthier dietary practices and increased physical activity. Similarly, Kandula (2016) [25] found that South Asian women participating in a culturally adapted group-based lifestyle intervention experienced favourable changes in HbA1c. Wieland (2024) [22] demonstrated meaningful reductions in HbA1c among East African immigrant adults with T2DM following a culturally grounded behavioural intervention delivered using trained community educators. Collectively, these studies indicate that culturally responsive lifestyle programmes can yield improvements in glycaemic outcomes among migrant populations at risk of T2DM.

### Fasting plasma glucose and other glycaemic indicators

Four studies assessed fasting plasma glucose or oral glucose tolerance, showing mixed but generally positive trends. Wikström et al. (2021) [23] reported a reduction in fasting glucose (−0.22 mmol/L) among Somali migrants who received culturally adapted lifestyle counselling, indicating favourable short-term metabolic shifts. In contrast, Kandula et al. (2016) [25] observed no significant change in fasting glucose despite improvements in diet quality scores, suggesting that diet-focused strategies alone may be insufficient without complementary physical activity components. Additional evidence from non-randomised and pre – post studies showed fasting glucose reductions ranging from −0.1 to −0.4 mmol/L [26–29]; however, these findings require cautious interpretation because the absence of control groups limits causal attribution. Overall, while the direction of effect across studies was generally favourable, variability in intervention content, intensity, and participant characteristics contributed to heterogeneity in outcomes and limits the ability to generalise findings or draw consistent conclusions across studies.

Table 2 summarises the five unique interventions with respect to cultural adaptations, health promotion principles and evaluation approach.

All five interventions included one or more culturally specific approaches. Bilingual deliverer was the most common approach reported in four interventions [22,23,25,26], three interventions included cultural competency training for staff [22,23,25], three interventions used culturally sensitive multimedia (e.g. video, figures, card) [22,23,25], two interventions used interpreters [25,26], and three interventions established community point-of-care [22,23,25]. Health promotion principles were missing in only one study [24]. In three studies, a co-design approach was implemented when relevant community members or organisations were involved in designing the interventions with the research staff [22,23,25], and in two interventions empowering principles or capacity-building approaches were implemented [23,24].

### Intervention dose

Programme durations varied: Kandula (2016) [25] and Melero (2020) [24] implemented shorter interventions (16 weeks and gestational weeks 8–38, respectively), while Telle-Hjellset (2013) [26] and Wieland (2024) [22] ran longer trials (7 and 6 months, respectively). In terms of dose, the most intensive intervention was exercise-focused involving 3 sessions a week that is 48 hours over the entire 4-month intervention [25], following by lifestyle education or counselling, which provided six sessions over the entire intervention period 12 hours contact with staff [26] or 9 hours contact [23]. The least intensive intervention was media based of 12-minute video and monthly prompts to watch [22].

Deliverers of interventions were health professionals whether exercise trainers, clinicians, dieticians or research staff with one intervention delivered by health sciences student volunteers. Adherence to interventions ranged from 50% [23] to as high as 89% [24]. Four studies included evaluation of the programmes, such as self-report satisfaction, interviews on experience with the programme, or indicators of antecedents to behaviour change.

### Quality appraisal findings

#### Randomised controlled trials

**Figure 2.**
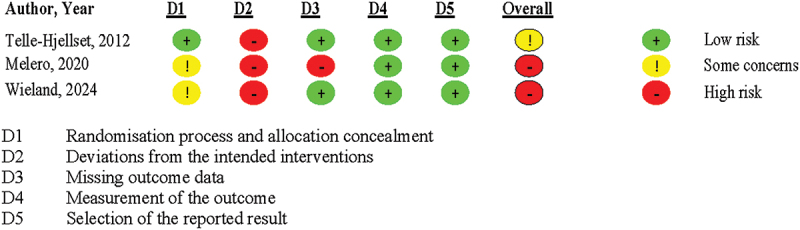
Quality appraisal using Cochrane risk of bias 2 (RoB2) tool across the five domains.

**Figure 3.**
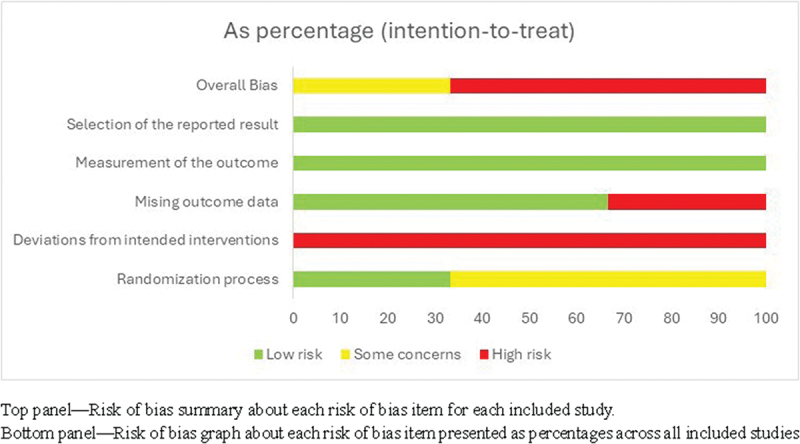
Risk of bias assessed for randomised trials.

The risks of bias varied across the included studies, with concerns primarily related to the randomisation process and allocation concealment, deviation from intended interventions and missing outcome data (Figure 2). While Telle-Hjellset (2013) [26] RCT demonstrated an appropriate randomisation method, Melero (2020) [24] and Wieland (2024) [22] RCTs lacked sufficient detail on allocation concealment, raising concerns about selection bias. Deviation from intended intervention were a major source of bias, as all trials lacked blinding and had inconsistencies in protocol adherence, potentially influencing the effectiveness of the interventions. Missing outcome data was another limitation with fewer studies experiencing loss to follow up without appropriate analytical methods to address it which could have affected the validity of the findings. In contrast, the risks of bias related to outcome measurement and selection of the reporting result was low (Figure 3). Overall, while the findings provide useful insights, the presence of high-risk domain in studies suggest that results should be interpreted with caution.

#### Non-randomised and pre–post studies

The two pre – post studies demonstrated variable methodological quality. Both lacked comparison groups and featured small sample sizes, which limited their internal validity. One study provided clear descriptions of intervention content and delivery, while the other reported limited detail, making replication difficult. Baseline characteristics were inconsistently reported, reducing comparability over time. Fidelity of intervention delivery was moderate, with attendance ranging between 50% and 70% where reported. Completeness of outcome data varied, with one study experiencing considerable attrition without appropriate adjustment. Potential risks of selection, performance, and detection bias were identified in both studies due to non-random allocation, self-reported outcomes, and limited blinding. Nevertheless, these studies contributed valuable insights into feasibility and acceptability of culturally tailored lifestyle interventions among migrant women.

*Certainty of evidence (GRADE*)

Using the GRADE approach, the certainty of evidence ranged from low to moderate across outcomes. Evidence for GDM incidence and physical activity outcomes was rated as *low certainty*, primarily due to imprecision, heterogeneity, and concerns about methodological limitations in several studies. HbA1c, fasting glucose, and dietary behaviour change outcomes were supported by *low – moderate to moderate certainty* evidence, with downgrades related to inconsistency and indirectness. Self-efficacy and motivation outcomes were rated as *low certainty* because of reliance on self-reported measures and small sample sizes. A full GRADE summary for all outcomes is presented in Supplementary Table S2.

Taken together, the evidence should be interpreted cautiously, as differences in study design, populations (including pregnancy status), intervention timing, and outcome measures mean that cross-study comparisons are inherently limited and conclusions cannot be generalised to all migrant populations or settings.

## Discussion

This systematic review highlights the limited availability of culturally adapted lifestyle interventions aimed at mitigating or preventing T2DM and GDM among migrant women from LMICs living in high-income countries. Only five unique studies were identified, including two pilot studies, indicating that research in this field remains in its early stages. Nevertheless, four of the five studies incorporated culturally tailored strategies, acknowledging the importance of cultural relevance in promoting healthy behaviours and improving glycaemic outcomes among migrant populations. The RCTs included in this review demonstrated moderate but promising effects on glycaemic outcomes, underscoring the potential of these interventions to address diabetes risk in this vulnerable population. However, the substantial heterogeneity across included studies spanning study designs, intervention timing (antenatal versus non-pregnancy), and outcome measures limits direct comparability and underscores the need for cautious interpretation of the overall evidence base.

The GRADE assessment indicated that the overall certainty of evidence ranged from low to moderate across outcomes, largely due to heterogeneity in study design, small sample sizes, and short follow-up durations. These findings highlight the need for more rigorous and adequately powered trials to strengthen confidence in the effectiveness of culturally tailored lifestyle interventions for migrant women from LMICs

Given the heterogeneity of outcomes across studies, GDM-specific outcomes (e.g. GDM incidence and gestational HbA1c) and T2DM-related metabolic outcomes (e.g. fasting glucose, insulin, HbA1c) were interpreted separately to avoid conceptual conflation. This distinction is important because GDM represents an immediate pregnancy-related condition, whereas T2DM reflects a longer-term metabolic trajectory that may be influenced by behaviours established both before and during pregnancy. By maintaining these analytical boundaries, the review reduces ambiguity while still recognising the shared behavioural and metabolic mechanisms underpinning both conditions.

Cultural and social norms play a significant role in shaping health behaviours among migrant women. Dietary patterns high in refined carbohydrates and saturated fats, coupled with gendered expectations that may limit physical activity, contribute to heightened diabetes risk. In addition, stressors related to migration including acculturation challenges, social exclusion, and discrimination can lead to adverse metabolic effects, further widening health disparities [30]. These intersecting factors underscore the need for interventions that not only educate and motivate behavioural change but also address broader systemic and sociocultural barriers.

To address health inequalities, delivering culturally appropriate care and interventions is essential. This approach involves respecting individuals’ beliefs, values, customs, knowledge, lifestyles, and social behaviours. Culturally responsive care ensures that interventions are tailored to the needs of diverse populations, ultimately empowering individuals to manage their health conditions more effectively [16,31].

In this study, the included interventions incorporated cultural components such as the use of bilingual deliverers, interpreter services, culturally sensitive multimedia, cultural competency training, and community point-of-care service engagement. This aligns with the findings of a systematic review by Henderson and See (2011) [16], which identified these five key categories of culturally sensitive interventions used to prevent chronic disease in general and emphasised the importance of integrating cultural awareness into healthcare strategies. Their review particularly supported the use of trained bilingual health workers with cultural competence as a critical factor in developing effective health service models for culturally and linguistically diverse (CALD) communities. Bilingual health workers that received culturally competence training were reported in three studies [21,23,25], but the diabetes-related outcomes were mixed, suggesting that more intensive strategies may be required among them boosting adherence, and conducting on-going process evaluation to identify barriers to change.

Lifestyle intervention with bilingual facilitators, culturally relevant education and the additional community engagement, such as Wikstrom (2021) [23] and Telle Hjelleset (2013) [26] showed significant improvements in glucose control and dietary behaviours. Despite Kandula (2016) [25] included these components, recorded limited metabolic impact, suggesting exercise intervention alone may be not sufficient without dietary intervention. Similarly, Melero (2020) [24], focusing on a Mediterranean diet intervention, demonstrated reduced GDM incidence though HBA1c showed moderate improvement. This is consistent with studies which demonstrated that combining physical activity and diet is more effective in lowering blood glucose level [32]. Wieland (2024) [22] utilised a digital storytelling and culturally tailored intervention programme. Although it showed small improvement of HbA1c, the scalability and portability make it an attractive intervention component for diabetes intervention due to lower access to health care and high acceptability and engagement.

In examining the relationship between cultural tailoring and observed glycaemic outcomes, our findings align with emerging evidence that the degree of cultural responsiveness influences both engagement and metabolic effectiveness. Interventions that incorporated multiple cultural components – such as bilingual facilitators, culturally adapted diet education, community-based delivery, and materials grounded in participants’ lived experiences – tended to report more favourable glycaemic outcomes. For example, interventions combining culturally relevant dietary modification with facilitated behaviour support demonstrated clearer improvements in fasting glucose and HbA1c, suggesting that alignment with cultural food practices increases feasibility and adherence. Conversely, exercise-only interventions showed limited metabolic benefit, emphasising that physical activity without dietary adaptation may be insufficient for meaningful glucose regulation among migrant women whose dietary norms require specific cultural contextualisation. Similarly, digital interventions demonstrated modest effects; while scalable and highly acceptable, their lighter-touch nature may not overcome structural and behavioural barriers faced by migrant women, such as competing caregiving responsibilities, limited health literacy, and need for personalised support. These patterns indicate that cultural tailoring is not only a mechanism for improving acceptability but may also directly influence metabolic outcomes by enhancing adherence, relevance, and sustained behaviour change.

Our findings broadly align with previous systematic reviews examining lifestyle interventions for diabetes prevention and management among migrant and ethnic minority populations. Rawal et al. (2021) [17] similarly reported that culturally adapted lifestyle interventions improved behavioural outcomes and modestly improved metabolic indicators among migrant and ethnic minority groups in high-income countries, although evidence quality remained limited. Meeks et al. (2016) [33] also highlighted substantial disparities in diabetes risk among ethnic minorities in Europe, underscoring the importance of culturally tailored strategies to address upstream social and structural drivers of dysglycaemia. In contrast, Sagastume et al. (2022) [10], who focused on lifestyle interventions implemented in LMICs, found more consistent improvements in cardiometabolic outcomes – likely reflecting greater intervention intensity and context-specific programme design. Consistent with these earlier reviews, our study reinforces the value of culturally responsive intervention components but also identifies a critical evidence gap specifically concerning migrant women from LMICs living in high-income settings, where unique sociocultural, structural, and migration-related barriers shape intervention effectiveness.

By raising awareness of the importance of culturally appropriate interventions and recognising studies that have successfully implemented such strategies, we emphasise the need for further research and policy development in this area. Ensuring that healthcare interventions are culturally responsive is a crucial step towards reducing health disparities and improving health outcomes among diverse and underserved populations.

## Study limitations

Despite the promising findings, several limitations must be acknowledged. First, the overall evidence base remains limited, with only five unique interventions identified across eight publications, including two pilot studies and several trials with small sample sizes and short follow-up periods. This narrow evidence base restricts the depth of synthesis and limits the generalisability of findings across diverse migrant subgroups. In addition, the included studies varied widely in intervention type, population characteristics, duration, delivery format, and outcome measurement, introducing considerable heterogeneity that constrained the ability to draw firm conclusions regarding overall effectiveness.

Second, methodological limitations related to study design, reporting quality, and risk of bias were common across the included studies. Among the randomised controlled trials, concerns were noted regarding allocation concealment, unblinded intervention delivery, deviations from intended protocols, and incomplete outcome data. Likewise, the pre – post studies lacked comparison groups, relied heavily on self-reported behavioural outcomes, and inconsistently reported baseline characteristics, limiting internal validity. Although these studies provided valuable insights into feasibility and acceptability, their designs reduced confidence in attributing the observed effects solely to the interventions. The GRADE assessment further reflected these weaknesses, with most outcomes rated as low to moderate certainty due to imprecision, inconsistency, and risk of bias.

Third, structural or contextual limitations related to the cultural, social, and community-engagement frameworks within which interventions were developed. This include the limited integration of co-design, community empowerment, and culturally grounded delivery models, factors that are essential for sustainable and contextually relevant interventions among migrant women from LMICs. Although all interventions incorporated at least one culturally tailored component, very few explicitly adopted co-design, community empowerment, or capacity-building principles – features that are essential for producing culturally resonant and sustainable health programmes. The absence of detailed reporting on implementation processes also limited the ability to understand which cultural components were most influential in driving behavioural or metabolic change.

Fourth, the search strategy was limited to peer-reviewed published studies and excluded grey literature, dissertations, and clinical trial registries. This may have resulted in publication bias, particularly given the likelihood that small or null-effect lifestyle interventions remain unpublished. Restricting the search to English-language publications may also have introduced language bias and limited representation of immigrant populations in non-English-speaking high-income countries.

Finally, several broader determinants of diabetes risk – including socioeconomic disadvantage, structural racism, healthcare access barriers, and migration-related stressors – were not consistently reported or controlled for, potentially confounding observed outcomes. These contextual factors are particularly salient for migrant women from LMICs and should be incorporated more rigorously into future intervention designs and analyses.

## Policy and practice implications and intervention scalability

The findings of this review have important implications for policy, practice, and the future scale-up of diabetes-prevention interventions for migrant women from LMICs in high-income countries. The consistent benefits observed in culturally tailored interventions, particularly those that integrated dietary modification, bilingual delivery, and community engagement, highlight the need for policies that prioritise culturally responsive care. Embedding cultural tailoring into routine maternal health and diabetes-prevention services, including the use of bilingual or bicultural health professionals and culturally adapted educational materials, is essential for improving engagement and outcomes. Antenatal care represents a critical entry point, offering an opportunity to integrate culturally adapted lifestyle counselling and strengthen coordination across obstetric, nutrition, and community health services.

The mixed metabolic outcomes across studies suggest that single-component interventions, such as digital-only or exercise-only models, are insufficient. Multi-component, culturally grounded strategies are more likely to drive meaningful glycaemic improvement, and policy frameworks should reflect this. Community-based and peer-supported models also show promise in enhancing accessibility and acceptability, indicating that partnerships with migrant community organisations are vital for long-term success.

Scaling these interventions will require system-level investment in a multicultural and culturally competent health workforce capable of delivering tailored care at larger scale. Although culturally anchored programmes may require greater initial resources, they are likely to deliver strong long-term value by improving adherence and preventing costly downstream complications. Scalability also depends on designing interventions with adaptable cultural components that can be applied across different migrant communities without full programme redesign. Integrating these programmes into existing health structures such as prenatal clinics and community health centres will further support sustainability.

Finally, expansion of such interventions must be guided by robust monitoring and evaluation systems that capture not only clinical outcomes but also culturally relevant measures of engagement and acceptability. Disaggregated data by migration background, ethnicity, and socioeconomic status are essential to ensure that scale-up efforts contribute to reducing, rather than reinforcing, existing health inequities.

## Future research

To strengthen the evidence base, future research should prioritise larger, adequately powered randomised controlled trials with long-term follow-up to assess whether behavioural and metabolic improvements are sustained beyond the immediate intervention period. Interventions must also be developed through meaningful engagement with migrant communities, using co-design, empowerment-oriented approaches, and culturally grounded delivery models. Greater attention to community capacity-building, structural barriers, and the lived realities of migrant women will enhance both cultural relevance and intervention sustainability. Additionally, future studies should clearly document the specific cultural components embedded within intervention design and evaluate how these mechanisms influence glycaemic and behavioural outcomes. Finally, the field would benefit from improved methodological consistency, standardised outcome measures, and transparent reporting to facilitate cross-study comparisons and evidence synthesis.

## Conclusion

This study provides a summary of the state of evidence of lifestyle interventions in reducing the risk of GDM and T2DM among migrant populations. The study underscores the paucity of interventions that are culturally tailored, community-based, and holistic to improving dietary habits, increasing physical activity, and reducing sedentary behaviour. The findings suggest that culturally adapted, multi-component lifestyle interventions show promise for improving behavioural and metabolic outcomes among migrant women from LMICs living in high-income settings. Interventions that integrate dietary modification, physical activity, and culturally responsive delivery approaches appear to be more effective than single-component strategies; however, the overall evidence base remains limited and characterised by variability in study design, populations, intervention timing, and outcome measures. As such, findings should be interpreted cautiously and within their specific clinical and methodological contexts, rather than generalised across settings or populations. It is also important to note that the narrative appraisal applied to non-randomised and pre – post studies, while structured, remains inherently subjective and lacks the standardisation of validated risk-of-bias tools; therefore, findings derived from these studies should be interpreted with additional caution. Further, high-quality, rigorously designed studies are needed to strengthen the evidence base and inform scalable, culturally appropriate interventions for this high-risk population.

## Supplementary Material

Supplementary File.docx

PRISMA checklist GHA REVISED.docx
