# Supplementary material for: Lifestyle interventions to prevent gestational and type 2 diabetes among migrant women from low- and middle-income countries: a systematic review
Source: Glob Health Action. 2026 Apr 17;19(1):2658925. doi: 10.1080/16549716.2026.2658925 (PMC13094288; doi:10.1080/16549716.2026.2658925)
Supplement: Supplementary File.docx [file ZGHA_A_2658925_SM4883.docx]

**Supplementary Table S1: Search strategy**

**PubMed: 19.12.2024**

| **ID** | **Query** | **Results** |
| --- | --- | --- |
|  | Pregnan*OR pregnancy [MeSH Terms:noexp] OR postpartum period [MeSH Terms:noexp] OR postpartum [Title/Abstract] OR post-partum [Title/Abstract] OR postnatal [Title/Abstract] OR post-natal [Title/Abstract] OR puerperium [Title/Abstract] OR mother* [Title/Abstract] OR maternal [Title/Abstract] OR women [Title/Abstract] OR woman[Title/Abstract]  AND  Low and middle income countr* [Title/Abstract] OR developing countr* [Title/Abstract] OR developing nation* [Title/Abstract] OR least developed countr* [Title/Abstract] OR under*developed countr* [Title/Abstract] OR under*developed nation* [Title/Abstract] OR LMIC* [Title/Abstract] OR low income countr* [Title/Abstract] OR middle income countr* [Title/Abstract] OR less-developed country [Title/Abstract:~6] OR less-developed countries [Title/Abstract:~6] OR less-developed nation [Title/Abstract:~6] OR less-developed nations [Title/Abstract:~6] OR third-world country [Title/Abstract:~6] OR third-world countries [Title/Abstract:~6] OR third-world nation [Title/Abstract:~6] OR third-world nations [Title/Abstract:~6] OR lower middle income country [Title/Abstract:~6] OR lower-middle-income-countries [Title/Abstract:~6] OR Developing Countries [MeSH Terms] OR Social Class [MeSH Terms] OR Emigrants and Immigrants [MeSH Terms] OR Transients and Migrants [MeSH Terms] OR emigrant* [Title/Abstract] OR immigra* [Title/Abstract] OR migrat* [Title/Abstract] OR migrant women [Title/Abstract] OR immigrant women [Title/Abstract] OR refugee* [Title/Abstract] OR foreigner* [Title/Abstract] OR migrant* [Title/Abstract] OR resident* [Title/Abstract]  AND  Life Style [MeSH Terms] OR life style* [Title/Abstract] OR lifestyle intervention* [Title/Abstract] OR lifestyle modification* [Title/Abstract] OR lifestyle counsel* [Title/Abstract] OR lifestyle program* [Title/Abstract] OR intervention* [Title/Abstract] OR prevention and control* [Title/Abstract] OR strateg* [Title/Abstract] OR effect* [Title/Abstract] OR risk reduc* [Title/Abstract] OR Diet [MeSH Terms:noexp] OR Diet, Carbohydrate-Restricted [MeSH Terms:noexp] OR Diet, Diabetic [MeSH Terms] OR Diet, Healthy [MeSH Terms] OR Diet, Reducing [MeSH Terms] OR Diet Therapy [MeSH Terms] OR diet* [Title/Abstract] OR supplement* [Title/Abstract] OR fibre supplement* [Title/Abstract] OR nutriti* [Title/Abstract] OR therap* [Title/Abstract] OR low carbohydrate diet [Title/Abstract] OR high* quality diet [Title/Abstract] OR low sugar diet [Title/Abstract] OR educat* [ Title/Abstract] OR counsel* [Title/Abstract] OR exercis* [Title/Abstract] OR physical activit* [Title/Abstract] OR exercise [MeSH Terms] OR walk* [Title/Abstract] OR workout [Title/Abstract] OR sport* [Title/Abstract] OR training [Title/Abstract] OR muscle strength* [Title/Abstract] OR resistance activit* [Title/Abstract] OR fitness [Title/Abstract] OR yoga [Title/Abstract] OR Aerobic* [Title/Abstract] OR jog [Title/Abstract] OR swim* [Title/Abstract] OR danc* [Title/Abstract]  AND  Diabetes, gestational [MeSH Terms] OR pregnancy diabetes mellitus [Title/Abstract] OR GDM [Title/Abstract] OR Pregnancy induced diabetes [Title/Abstract] OR diabetes mellitus, type 2 [MeSH Terms:noexp] OR gestational diabet* [Title/Abstract] OR type 2 diabet* [Title/Abstract] OR type 2 diabetes mellitus [Title/Abstract] OR T2DM [ Title/Abstract] OR non-insulin dependent diabetes mellitus [Title/Abstract] | 1042 |

**Embase: 19.12.2024**

| **ID** | **Query** | **Results** |
| --- | --- | --- |
| #1 | Embase Classic+Embase <1947 to 2024 December 17>  Pregnan* OR postnatal OR post-natal OR maternal OR mother* OR woman OR women OR postpartum OR postpartum period OR post-partum OR postnatal care OR puerperium | 3105142 |
| #2 | migrant* OR immigrant* OR emigrant* OR foreigner* OR resident* OR refugee OR immigrant women OR migrant women OR migrat* OR social class OR low-and middle-income countr* OR developing countr* OR developing nation* OR least developed countr* OR under*developed countr* OR under*developed nation* OR LMIC* OR low income countr* OR middle income countr* | 1117775 |
| #3 | Life style* OR lifestyle intervention* OR lifestyle modification* OR lifestyle counsel* OR lifestyle program* OR intervention* OR prevention and control* OR strateg* OR effect* OR risk reduc* OR diet* OR supplement* OR fibre supplement* OR nutriti* OR therap* OR low carbohydrate diet OR high* quality diet OR low sugar diet OR educat* OR counsel* OR exercis* OR physical activit* OR walk* OR workout OR sport* OR training OR muscle strength* OR resistance activit* OR fitness or yoga or Aerobic* or jog* or swim* or danc* | 19067093 |
| #4 | GDM OR gestational diabet* OR Pregnancy induced diabetes OR diabetes mellitus OR type 2 diabet* OR T2DM | 342588 |
| #5 | #1 AND #2 AND #3 | 1675 |

**Scopus: 19.12.2024**

| **ID** | **Query** | **Results** |
| --- | --- | --- |
| #1 | TITLE-ABS-KEY (pregnan* OR postnatal OR post-natal OR postpartum OR mother* OR "postpartum period" OR puerperium OR maternal OR woman OR women)  AND  TITLE-ABS-KEY (migrant* OR immigrant* OR emigrant* OR foreigner* OR migrat* OR "migrant women" OR "migrant woman" OR refugee OR "social class" OR "low-and middle-income countr*" OR "developing countr*" OR "developing nation*" OR "least developed countr*" OR "under*developed countr*" OR "under*developed nation*" OR LMIC* OR "low income countr*" OR "middle income countr*”)  AND  TITLE-ABS-KEY (diet* OR counsel* OR nutrit* OR "nutrition education" OR "fibre supplement*" OR "low carbohydrate diet" OR "diabetic diet" OR "diet restrict*" OR "diet therap*" OR "diet reduc*" OR "high* quality diet" OR "low sugar diet" OR exercise* OR "physical activit*" OR "walk*" OR "muscle strength*" OR train* OR "resistance activit*" OR yoga OR sport* OR swim* OR workout OR "aerobic activit*" OR intervention* OR prevention OR strateg* OR effective* OR " lifestyle intervention*" OR "lifestyle modification*" OR reduc* OR "risk reduc*" OR "lifestyle*" OR "lifestyle induced illness*" OR "lifestyle factor*" OR "life style" )  AND  TITLE-ABS-KEY (“gestational diabet*" OR "type 2 diabet*" OR "gestational diabetes mellitus" OR "pregnancy-induced diabet*" OR "type 2 diabetes mellitus" OR GDM OR T2DM OR "maternal diabetes mellitus" OR "pregnancy diabetes mellitus" OR "non-insulin dependent diabetes mellitus" OR "type 2 diabetes") | 1260 |

**CINAHL: 19.12.2024**

| **ID** | **Query** | **Results** |
| --- | --- | --- |
| S1 | TI ( pregnan* or postnatal or post-natal or maternal or mother* or woman or women or postpartum or "postpartum period" or "post-partum" or "postnatal care" or puerperium ) OR AB ( pregnan* or postnatal or post-natal or maternal or mother* or woman or women or postpartum or "postpartum period" or "post-partum" or "postnatal care" or puerperium ) OR SU ( pregnan* or postnatal or post-natal or maternal or mother* or woman or women or postpartum or "postpartum period" or "post-partum" or "postnatal care" or puerperium) | 181 |
| S2 | (MH "Pregnancy") |  |
| S3 | (MH "Postnatal Care") |  |
| S4 | (MH "Postnatal Period") OR (MH "Puerperium") |  |
| S5 | TI (migrant* or immigrant* or emigrant* or foreigner* or resident* or refugee or "immigrant women" or "migrant women" or migrat* or "social class" or "low-and middle-income countr*" or "developing countr*" or "developing nation*" or "least developed countr*" or "under*developed countr*" or "under*developed nation*" or LMIC* or "low income countr*" or "middle income countr*" ) OR AU ( migrant* or immigrant* or emigrant* or foreigner* or resident* or refugee or "immigrant women" or "migrant women" or migrat* or "social class" or "low-and middle-income countr*" or "developing countr*" or "developing nation*" or "least developed countr*" or "under*developed countr*" or "under*developed nation*" or LMIC* or "low income countr*" or "middle income countr*" ) OR SU ( migrant* or immigrant* or emigrant* or foreigner* or resident* or refugee or "immigrant women" or "migrant women" or migrat* or "social class" or "low-and middle-income countr*" or "developing countr*" or "developing nation*" or "least developed countr*" or "under*developed countr*" or "under*developed nation*" or LMIC* or "low income countr*" or "middle income countr*") |  |
| S6 | (MH "Immigrants") OR (MH "Migrants") OR (MH "Emigration and Immigration") |  |
| S7 | (MH "Refugees") |  |
| S8 | TI ( "life style*" or "lifestyle intervention*" or "lifestyle modification*" or "lifestyle counsel*" or "lifestyle program*" or intervention* or "prevention and control*" or strateg* or effect* or "risk reduc*" or diet* or supplement* or "fibre supplement*" or nutriti* or therap* or "low carbohydrate diet" or "high* quality diet" or "low sugar diet" or "educat*" or counsel* or exercis* or "physical activit*" or walk* or workout or sport* or training or "muscle strength*" or "resistance activit*" or fitness or yoga or Aerobic* or jog* or swim* or danc* ) OR AB ( "life style*" or "lifestyle intervention*" or "lifestyle modification*" or "lifestyle counsel*" or "lifestyle program*" or intervention* or "prevention and control*" or strateg* or effect* or "risk reduc*" or diet* or supplement* or "fibre supplement*" or nutriti* or therap* or "low carbohydrate diet" or "high* quality diet" or "low sugar diet" or "educat*" or counsel* or exercis* or "physical activit*" or walk* or workout or sport* or training or "muscle strength*" or "resistance activit*" or fitness or yoga or Aerobic* or jog* or swim* or danc* ) OR SU ( "life style*" or "lifestyle intervention*" or "lifestyle modification*" or "lifestyle counsel*" or "lifestyle program*" or intervention* or "prevention and control*" or strateg* or effect* or "risk reduc*" or diet* or supplement* or "fibre supplement*" or nutriti* or therap* or "low carbohydrate diet" or "high* quality diet" or "low sugar diet" or "educat*" or counsel* or exercis* or "physical activit*" or walk* or workout or sport* or training or "muscle strength*" or "resistance activit*" or fitness or yoga or Aerobic* or jog* or swim* or danc* ) |  |
| S9 | (MH "Dietary Supplementation") OR (MH "Nutritional Counseling") OR (MH "Nutrition") OR (MH "Dietary Supplements") |  |
| S10 | (MH "Life Style, Sedentary") OR (MH "Life Style") OR (MH "Life Style Changes") OR (MH "Health Behavior") |  |
| S11 | (MH "Counseling") OR (MH "Nutrition Services") OR (MH "Nutritional Counseling") |  |
| S12 | (MH "Nutrition Education") |  |
| S13 | (MH "Counseling") OR (MH "Nutritional Counseling") OR (MH "Nutrition Services") |  |
| S14 | (MH "Exercise") OR (MH "Endurance Training") OR (MH "High-Intensity Interval Training") OR (MH "Warm-Up Exercise") OR (MH "Exercise Intensity") OR (MH "Physical Endurance") OR (MH "Aerobic Exercises") OR (MH "Group Exercise") OR (MH "Lower Extremity Exercises") OR (MH "Muscle Strengthening") OR (MH "Stretching") OR (MH "Walking") OR (MH "Physical Activity") OR (MH "Sports") |  |
| S15 | (MH "Diabetic Diet") OR (MH "Diet, Reducing") OR (MH "Restricted Diet") OR (MH "Diet") OR (MH "Diet, Low Carbohydrate") |  |
| S16 | TI ( GDM or "Pregnancy induced diabetes" or "gestational diabet*" or "type 2 diabet*" or T2DM or "non insulin dependent diabetes mellitus" or "diabetes mellitus type 2" or "pregnancy diabetes mellitus" or "maternal diabetes mellitus" ) OR AU ( GDM or "Pregnancy induced diabetes" or "gestational diabet*" or "type 2 diabet*" or T2DM or "non insulin dependent diabetes mellitus" or "diabetes mellitus type 2" or "pregnancy diabetes mellitus" or "maternal diabetes mellitus" ) OR SU ( GDM or "Pregnancy induced diabetes" or "gestational diabet*" or "type 2 diabet*" or T2DM or "non insulin dependent diabetes mellitus" or "diabetes mellitus type 2" or "pregnancy diabetes mellitus" or "maternal diabetes mellitus" ) |  |
| S17 | (MH "Pregnancy in Diabetes") OR (MH "Diabetes Mellitus, Gestational") |  |
| S18 | (MH "Diabetes Mellitus, Type 2") |  |
| S19 | S1 OR S2 OR S3 OR S4 |  |
| S20 | S5 OR S6 OR S7 |  |
| S21 | S19 AND S20 |  |
| S22 | S8 OR S9 OR S10 OR S11 OR S12 OR S13 OR S14 OR S15 |  |
| S23 | S16 OR S17 OR S18 |  |
| S24 | S21 AND S22 AND S23 |  |

**The Cochrane central: 19.12.2024**

| **ID** | **Query** | **Results** |
| --- | --- | --- |
| #1 | Pregnan* OR postnatal OR post natal OR maternal OR mother* OR woman OR women OR postpartum OR "postpartum period" OR "post partum" OR "postnatal care" OR puerperium | 259107 |
| #2 | MeSH descriptor: [Pregnancy] this term only | 34209 |
| #3 | MeSH descriptor: [Postpartum Period] this term only | 2126 |
| #4 | MeSH descriptor: [Postnatal Care] this term only | 601 |
| #5 | #1 OR #2 OR #3 OR #4 | 259107 |
| #6 | (migrant* OR immigrant* OR emigrant* OR foreigner* OR resident* OR refugee OR "immigrant women" OR "migrant women" OR migrat* OR "social class"): ti,ab,kw | 26041 |
| #7 | MeSH descriptor: [Transients and Migrants] this term only | 136 |
| #8 | MeSH descriptor: [Emigrants and Immigrants] explode all trees | 311 |
| #9 | (low and middle income NEXT countr*): ti,ab,kw OR (developing NEXT countr*): ti,ab,kw OR (developing NEXT nation*): ti,ab,kw OR (least developed NEXT countr*): ti,ab,kw OR (underdeveloped NEXT countr*): ti,ab,kw | 7932 |
| #10 | (under developed NEXT nation*):ti,ab,kw OR (LMIC*):ti,ab,kw OR (low income NEXT countr*) OR (middle income NEXT countr*) | 4453 |
| #11 | #6 OR #7 OR #8 OR #9 OR #10 | 34838 |
| #12 | #5 AND #11 | 6288 |
| #13 | (life NEXT style*):ti,ab,kw OR (lifestyle NEXT intervention*):ti,ab,kw OR (lifestyle NEXT modification*):ti,ab,kw OR (lifestyle NEXT counsel*):ti,ab,kw OR (lifestyle NEXT program*):ti,ab,kw | 16814 |
| #14 | intervention* OR strateg* OR effect* OR diet* OR supplement* OR nutriti* OR therap* OR "low carbohydrate diet" OR "higher quality diet" OR "low sugar diet" OR educat* OR counsel* OR exercis* OR walk* OR workout OR sport* OR training OR fitness OR yoga OR Aerobic* OR jog* OR swim* OR danc*):ti,ab,kw | 1840256 |
| #15 | (resistance NEXT activit*):ti,ab,kw OR (prevention NEXT control*):ti,ab,kw OR (risk NEXT reduc*):ti,ab,kw OR (fibre NEXT supplement*):ti,ab,kw OR (physical NEXT activit*):ti,ab,kw | 188062 |
| #16 | (muscle NEXT strength*): ti,ab,kw | 24400 |
| #17 | MeSH descriptor: [Life Style] this term only | 5189 |
| #18 | MeSH descriptor: [Diet] this term only | 10840 |
| #19 | MeSH descriptor: [Diet Therapy] this term only | 492 |
| #20 | MeSH descriptor: [Exercise] this term only | 26207 |
| #21 | #13 OR #14 OR #15 OR #16 OR #17 OR #18 OR #19 OR #20 | 1848894 |
| #22 | (GDM or "Pregnancy induced diabetes" OR T2DM OR "non insulin dependent diabetes mellitus" OR "diabetes mellitus type 2" OR "pregnancy diabetes mellitus" OR "maternal diabetes mellitus"):ti,ab,kw OR (gestational NEXT diabet*):ti,ab,kw OR (type 2 NEXT diabet*):ti,ab,kw | 61061 |
| #23 | MeSH descriptor: [Diabetes, Gestational] this term only | 1697 |
| #24 | MeSH descriptor: [Diabetes Mellitus, Type 2] this term only | 27016 |
| #25 | #22 OR #23 OR #24 | 61138 |
| #26 | #12 AND #21 AND #25 | 173 |

**Supplementary Table S2: Summary of Certainty of Evidence (GRADE)**

| **Outcome** | **Study Designs Contributing Evidence** | **Summary of Findings** | **GRADE Domains** | **Overall Certainty of Evidence** |
| --- | --- | --- | --- | --- |
| **GDM incidence** | 2 RCTs, 1 pre–post intervention | Lifestyle interventions showed mixed effects; one study reported reduced GDM incidence while others showed no significant difference. | **Risk of bias:** Some concerns in RCTs; serious concerns in non-RCTs. **Inconsistency:** Moderate heterogeneity. **Indirectness:** Low. **Imprecision:** Serious (small samples). **Publication bias:** Unclear. | **Low certainty** |
| **HbA1c (glycated haemoglobin)** | 3 RCTs, 1 pilot RCT, 1 pre–post study | Small to moderate decreases in HbA1c across most studies; effects more evident in interventions combining diet + physical activity. | **Risk of bias:** Some concerns. **Inconsistency:** Low–moderate. **Indirectness:** Low. **Imprecision:** Serious due to small samples. **Publication bias:** Possible. | **Moderate certainty** |
| **Fasting glucose / glucose control** | 3 RCTs, 1 non-RCT | Culturally tailored interventions improved fasting glucose in two studies; limited or no effect in interventions using physical activity alone. | **Risk of bias:** Some concerns. **Inconsistency:** Moderate (differences in intervention type). **Indirectness:** Low. **Imprecision:** Serious (pilot-scale studies). **Publication bias:** Possible. | **Low–moderate certainty** |
| **Dietary behaviour change** | 4 studies (RCTs + quasi-experimental + pre–post) | Consistent improvement in dietary knowledge, reduced intake of refined carbohydrates, and increased healthy eating practices. | **Risk of bias:** Some concerns. **Inconsistency:** Low. **Indirectness:** Low. **Imprecision:** Moderate. **Publication bias:** Possible. | **Moderate certainty** |
| **Physical activity behaviour change** | 3 studies | Improvements reported mainly in interventions combining PA with diet; PA-only interventions showed weaker effects. | **Risk of bias:** Some concerns. **Inconsistency:** Moderate. **Indirectness:** Low. **Imprecision:** Serious (limited sample size). **Publication bias:** Unclear. | **Low certainty** |
| **Self-efficacy / Motivation / Knowledge** | 2 studies | Increased motivation and diabetes-related knowledge reported, particularly where bilingual health workers or cultural tailoring were included. | **Risk of bias:** Serious concerns (self-reported outcomes). **Inconsistency:** Low. **Indirectness:** Moderate (proxy outcomes). **Imprecision:** Serious. **Publication bias:** Unclear. | **Low certainty** |
